# The Effect of Stimulus Size and Eccentricity on Attention Shift Latencies

**DOI:** 10.3390/vision1040025

**Published:** 2017-12-07

**Authors:** Louisa Kulke

**Affiliations:** 1 Department of Affective Neuroscience and Psychophysiology, Göttingen University, Leibniz Science Campus Primate Cognition, 37073 Göttingen, Germany; lkulke@uni-goettingen.de; Tel.: +49-(0)551-39-20624; 2 Division of Psychology and Language Sciences, Faculty of Brain Sciences, University College London, London WC1E 6BT, UK

**Keywords:** attention, fixation shift paradigm, gap–overlap paradigm, infancy, eye-tracking

## Abstract

The ability to shift attention between relevant stimuli is crucial in everyday life and allows us to focus on relevant events. It develops during early childhood and is often impaired in clinical populations, as can be investigated in the fixation shift paradigm and the gap–overlap paradigm. Different tests use stimuli of different sizes presented at different eccentricities, making it difficult to compare them. This study systematically investigates the effect of eccentricity and target size on refixation latencies towards target stimuli. Eccentricity and target size affected attention shift latencies with greatest latencies to big targets that were presented at a small eccentricity. Slowed responses to large parafoveal targets are in line with the idea that specific areas in the superior colliculus can lead to inhibition of eye movements. Findings suggest that the two different paradigms are generally comparable, as long as the target is scaled in proportion to the eccentricity.

## 1. Introduction

Eye movements can provide valuable insights into cognitive processes, including visual attention. Particularly in infants and non-verbal populations, eye movement can be used to study processes without the use of verbal instructions (e.g., [1,2]). The ability to shift gaze between stimuli is an established measure of development and can be used as a predictor for developmental outcomes in clinical populations for example in children with Williams Syndrome [3], pre-term born infants [4], siblings of autistic children [5,6], infants with hypoxic-ischaemic encephalopathy (HIE) [7,8] and children who had one of their brain’s hemispheres removed (hemispherectomised children, [9]). However, it is unclear how the visual features of stimuli used to measure attention shifts affect the overall findings. Two well-established behavioural methods, originally designed to examine overt shifts of attention in young infants, are the fixation shift paradigm (FSP, e.g., [10,11,12,13,14,15,16,17]) and the gap–overlap paradigm (e.g., [5,18,19,20,21,22]). In the fixation shift paradigm infants are shown one stimulus centerd on a screen for a short period of time. When the infant fixates the central target, a second one is presented in another location—either while the first stimulus is still present (competition condition), or immediately after the first stimulus disappears (non-competition condition). The gap paradigm introduces an additional condition in which the second stimulus appears after the first stimulus has been turned off for a certain time (gap condition, e.g., [12,19,20,23,24]). Differences in refixation latencies between competition and non-competition conditions have been demonstrated reliably in different setups using both of these paradigms in typically developing and clinical populations (e.g., [11,12,19,25]). Despite developmental changes in the competition condition, clear differences between competition and non-competition conditions can be observed in healthy infants [15] and adults [26,27], making these tasks suitable to test attention shifts across the life span.

Although the two paradigms are very similar in structure and conditions they use, they differ only in regards to their stimulus features. The originally used stimuli in the fixation shift paradigm are a central schematic face and large peripheral high contrast bars, being well above the acuity threshold and therefore particularly suitable for young infants [11,12]. However, the stimuli used in the similar gap–overlap paradigm have often differed in size and eccentricity from the fixation shift paradigm stimuli. For example, Csibra, Johnson and Tucker [27] used a gap paradigm task to monitor ERPs during fixation shifts; their stimuli had a size of 0.33° and were displayed at a eccentricity of 5°. However, stimuli modelled on the fixation shift paradigm (e.g., [12]), have been higher contrast, bigger (3.1° × 13.2°) and presented at a greater eccentricity (12.9°) [15,28]. The aim of the current study was to investigate potential differences between the two paradigms by directly comparing saccade latencies to stimuli with the sizes and eccentricities used in the original clinically used paradigms.

Previous literature directly comparing the effect of stimulus eccentricity in one experimental setup is rare and most studies investigating visual attention effects used manual responses rather than eye-movement latencies as an outcome measure (e.g., [29,30,31]). To our best knowledge, no previous research used stimuli with features comparable to clinically used paradigms. However, studies using unrelated stimuli suggest that eye movements may depend on stimulus size and eccentricity. An early study used electroocculograms (EOG) to investigate differences between saccades to LED lights at different eccentricities and manually scored saccade onset [32]. They found eye movement latency to increase with target eccentricity but only at low target intensities, which are rarely used in clinical studies as the targets need to be well above patients’ or infants’ acuity threshold. However, at high target intensities no such effect was found. Thanks to technological advances in eye tracking, nowadays automated methods can be used to track eye movements (e.g., [1,2]). In an infrared eye tracking study, Hodgson [33] showed that eye movement latencies increase with target eccentricity but only if the target location is marked using location markers. In a systematic investigation of the effect of different target sizes and eccentricities on eye movement latencies, Dick et al. [34] found that fixation shift latencies can significantly vary depending on the eccentricity at which the target stimulus is presented. However, stimulus size only had a significant effect on eye movement latency at small eccentricities under 10 degree of visual angle, which previously have mainly been used in the gap–overlap paradigm (e.g., [27]) but not in the fixation shift paradigm. In specific, large targets close to foveal areas seem to lead to longer saccade latencies. Visual perception is considerably more sensitive in the fovea than in the periphery, related to larger cortical areas associated with processing of foveal information [35,36] and smaller receptive field size [36], making the fovea more sensitive to high spatial frequencies. However, large stimuli close to the fovea might have inhibitory effects on saccades. Visual stimuli can activate the superior colliculus (SC) [37,38], a structure involved in eye movement execution and suppression [37,39]. Dick, Ostendorf, Kraft, and Ploner [34] suggest that centrally presented targets within 10 degree of the fovea may activate parts of the SC that inhibit rather than initiate eye movements, leading to longer latencies.

The SC is interconnected with areas related to attention control. Visual responses increase due to attention allocation [40,41] and have been connected to attention shifting in the fixation shift paradigm [16,17]. MRI research investigating attention effects in the visual cortex as a function of the distance between two stimuli showed that attention effects depend on receptive field size of the areas processing the respective stimulus [31]. Distance did not affect attention in areas with small receptive fields (i.e., Va, V2 and VP), but in areas with larger receptive fields (V4). Although visual areas were found to be crucial for attention shifts in EEG studies [16,17,27], it is unclear which exact areas underlie the observed EEG responses and, therefore, whether the distance between center and target affects attentional responses in the current paradigm. In addition to its interconnectedness with visual cortical areas, SC is further integrated in neuronal attention networks including the prefrontal cortex [42], involved in explicit control of eye-movements [39,43,44,45,46,47,48,49] and target selection [37], and the frontal eye-fields involved in attentional selection (review [50]). Visual stimuli can effect processing throughout the neural networks of attention and early visual responses have been linked to attention shifting in the fixation shift paradigm [16,17]. Therefore, the visual features of stimuli used for attention shifting tasks might potentially affect both the overt attention shifting behavior, measured through eye-movements, as well as neural processes underlying attention. In summary, previous literature shows that target eccentricity and size can affect eye movement latencies; however, this effect strongly depends on stimulus features and context and can therefore not be generalized to the stimulus types used in clinical and attention studies.

The current study aimed to investigate potential differences in fixation shift latencies in response to stimuli of different sizes and eccentricities used in clinical research to test whether these stimulus properties may affect findings and comparison between different studies. It was conducted with adult participants, as they show similar effects in the task as infants according to previous research [26,27], but can be instructed more easily leading to less noise in the data and therefore more power to detect potential differences between the paradigms. In general, the eye-tracking methodology is suitable for both infants and adults (for a review, see [2]). It was hypothesised that similar refixation latencies can be found using different stimulus types.

## 2. Results

Full datasets are provided in supplementary material. A repeated measures ANOVA was used to analyze the effect on refixation latency of target size, eccentricity and their interaction. Means and standard deviations of refixation latency (in ms) for the different conditions are displayed in Table 1.

There were no significant main effects of target size, *F*(1, 21) = 0.16, *p* = 0.694, η_p_^2^ = 0.008, but a small effect of eccentricity, *F*(1, 21) = 4.48, *p* = 0.046, η_p_^2^ = 0.176, and a significant interaction effect of target size and eccentricity, *F*(1, 21) = 12.44, *p* < 0.001, η_p_^2^ = 0.372.

Further analyses using dependent samples t-tests showed that for a target size of 3.1° × 13.2°, participants reacted significantly faster to targets at an eccentricity of 12.9° than at 5°, *t*(21) = 3.44, *p* = 0.002, *d* = 1.47. Eccentricity did not have a significant effect on refixation latency when the stimulus was a 0.33° square, *t*(21) = −0. 32, *p* = 0.754, *d* = 0.14. Latency was significantly shorter for small than for large targets at an eccentricity of 5°, *t*(21) = −2.31, *p* = 0.031, *d* = 0.99, but significantly longer for small than for large targets at an eccentricity of 12.9°, *t*(21) = 2.48, *p* = 0.022, *d* = 1.06. Crucially, there was no significant difference in latency between small targets at small eccentricities (comparable to Csibra et al. [27]) and lager targets at large eccentricities (comparable to Kulke et al. [15]), t(21) = 1.79, p = 0.088, *d* = 0.76.

Follow up Bayesian analyses were conducted using the anovaBF function of the “BayesFactor” Package [51] in R [52] using Cauchy priors based on Liang et al. [53]. The full factorial model with target size and eccentricity as within-participant factors revealed that it is 4.2 times more likely that there is no effect of target size than that there is one (*BF*_10_ = 0.236), that it is 2.6 times more likely that there is an effect of eccentricity than that there is none (*BF*_10_ = 2.587). A follow up analysis of the interaction effect of target size and eccentricity compared a full factorial linear model with a model excluding the interaction effect and showed that an interaction effect is 8.9 times more likely than no interaction (*BF*_10_ = 8.855). A comparison of latency between small targets at small eccentricities (comparable to Csibra et al. [27]) and lager targets at large eccentricities (comparable to Kulke et al.) showed that it is 1.2 times more likely that there is no difference than that there is one (*BF*_10_ = 0.871).

## 3. Discussion

There was no significant difference in refixation latency between targets matching the gap–overlap paradigm (Csibra et al. [27]) and the fixation shift paradigm (Kulke et al. [15]) stimuli in size and eccentricity and Bayesian analyses confirm that these differences are unlikely to affect refixation latency. This is in line with the hypothesis that the target features commonly used in infant attention research only have a negligible effect on refixation latencies and supports previous literature showing no effects when high-intensity stimuli are used without any contextual markers [32,33]. However, there was an effect of target eccentricity as well as a significant interaction of target size and eccentricity, showing that subjects responded more slowly to the big target stimulus when it was closer to the center of the screen. This interaction is in line with findings from Dick, Ostendorf, Kraft, and Ploner [34], who also found that large stimuli close to the fovea elicit slower eye movements. For their study, Dick, Ostendorf, Kraft and Ploner [34] used controlled stimuli that only differed in the degree of visual angle they covered, while the size of stimuli used in the current study differed in both the degree of visual angle, as well as the shape (square or rectangle) to allow for direct comparison with clinically used paradigms. The findings show that similar effects occur independent of shape. It should be noted that the current study compared the original stimulus types used for two major infant paradigms. Although the findings are in line with literature controlling further stimulus properties [32,33,34], the stimuli were based on their clinical relevance rather than controlled properties in the current study and complement previous research using detailed manipulations of stimulus properties.

At first sight, it might seem counter-intuitive that responses were decelerated towards large foveal stimuli, as visual processing is more fine-grained at the fovea than in the periphery [35,36], which should facilitate responses. However, for the eye-movements measured in the current study, SC plays a crucial role, being involved in both their execution and suppression [37,39]. It is possible that large stimuli close to the fovea activate inhibitory circuits within the SC, leading to a delay of eye movements [34]. This provides further support to the idea that large centrally presented stimuli can inhibit eye movements. As SC is interconnected with areas involved in attention control [42,54,55], the different activation pattern due to large targets close to the fovea might in turn also affect attentional neural responses. Therefore, large foveal targets should be avoided to ensure comparability of attention paradigms.

Alternatively, the longer latencies may be due to an implicit tendency to look at the center of a target stimulus [56,57]. For a large target of small eccentricity, subjects may have more angular uncertainty about the center of the target, making it more difficult to compute the direction they should make the eye movement in. Similarly, subjects may need additional processing time for the decision, which part of the objects they will look at and might be covertly exploring the target before the overt shift of attention. Longer saccadic latencies for large foveal targets may therefore be due to the greater variety of possibilities which location within the target the eye-movement will be directed to, leading to additional processing time required for the decision. Previous research by Ploner et al. [58] shows that saccade amplitudes are more scattered for larger than for smaller targets, being in line with both of these interpretations. Based on the current study it can therefore be recommended to use smaller stimuli at small eccentricities and bigger stimuli at greater eccentricities, i.e., to scale the stimulus in proportion to the eccentricity when designing attention shift tasks.

The cortical magnification theory suggests that stimuli are perceived similarly across the visual field as long as their cortical representations are comparable, i.e., they are scaled using a cortical magnification factor accounting for difference between fovea and periphery (e.g., [59,60], see [61] for an overview). Large peripheral and small central targets should induce similar cortical representations once the magnification factor is applied, leading to comparable findings for these types of stimuli in the current study. Additionally, early research by Yeshurun and Carrasco [62] suggests that attention enhances spatial resolution, improving processing of peripheral stimuli with low spatial resolution, but impairing processing of stimuli with high spatial resolution close to the fovea, as the attentional filter enhancing resolution might be too high to fully grasp the texture. This might result in large objects close to the fovea being more difficult to process in the current study.

As both the fixation shift paradigm and the gap–overlap paradigm previously showed clear differences between competition and non-competition conditions in both infants [15] and adults [26,27], only adult subjects were tested in the current study. Presuming that adult data is less noisy and provides more power to detect potential differences between the paradigms, it is unlikely that a significant difference between paradigms would be present in infants. However, perceptual mechanisms may differ between infants and adults; therefore, future research should explore whether both paradigms are comparable in an infant sample.

The current study shows that stimuli that are commonly used to study attention using the fixation shift paradigm (big size, great eccentricity) elicit eye movements at comparable latencies to the stimuli commonly used in the gap–overlap task (small size, small eccentricity). The observed effects in both paradigms can therefore be compared independent of the size and eccentricity of stimuli they use. In a clinical context, this is relevant as both paradigms have been used in clinical and infant populations, as a tool to detect developmental delays ([3,4,5,6,7,8]) and the current study suggests that the results, at least with the commonly used targets, are comparable between studies.

## 4. Materials and Methods

### 4.1 Participants

Twenty-two healthy adults (*M*_age_ = 20.09 years, *SD* = 1.35, 8 male) with normal or corrected to normal vision volunteered to participate in the study after informed written consent was obtained. A sample size calculation using G*Power software [63] showed that, based on the original effect size *f* = 2.39 in a previous gap–overlap paradigm study in adults [27], a minimum sample size of seven participants would be sufficient to detect the previously observed effects. The sample size in the current study was chosen well above this minimum required sample size. The study was approved by the University College London (UCL) Research Ethics Committee (Project ID Number: CPB/2014/007) and conducted according to the World Medical Association Declaration of Helsinki.

### 4.2 Design and Stimuli

Stimulus shapes, sizes, and eccentricities were directly based on the original fixation shift paradigm and the original gap–overlap paradigm stimuli. In a 2×2 factorial design the effect of target size on refixation latencies towards a target were measured using Csibra et al.’s gap–overlap paradigm targets (0.33° square [27]) or Kulke et al.’s fixation shift paradigm target [15], a 3.1° × 13.2° rectangle [15,16,28]. Eccentricity of the target (defined with respect to the center of each target) was either 5° (Csibra et al. [27]) or 12.9° (Kulke et al. [15]) from the center of the screen. MATLAB7.11.0 (R2010b) was used to generate the stimuli on a CRT monitor (Samsung). A Tobii X120 eye tracker monitored eye movements of participants at a sampling rate of 60 Hz. Only overlap conditions were used as they are the primary indicator of development with age [15] and relevant in clinical settings [13]. Fuller details of the configuration and procedure are described in Kulke, Atkinson, and Braddick [15].

**Figure 1:**
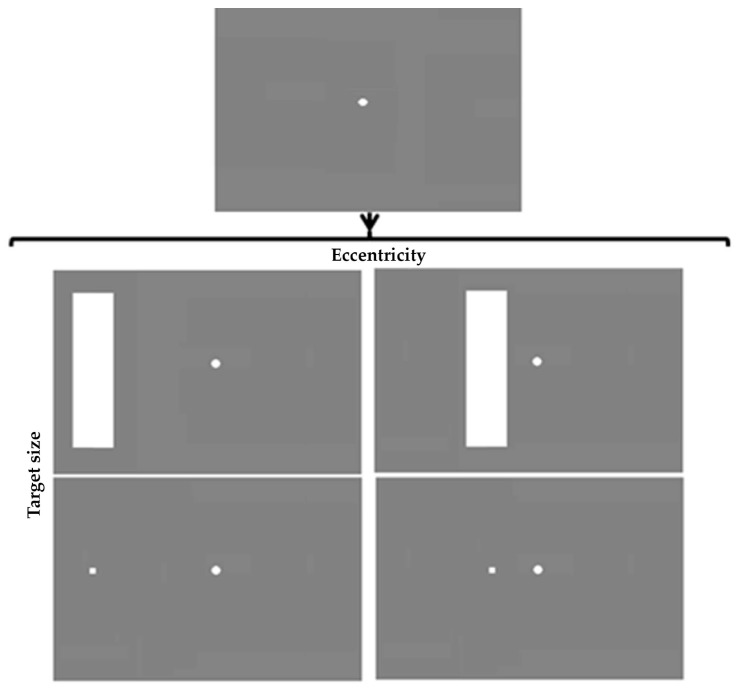
Conditions differ in target size and eccentricity.

Conditions were completed in four separate blocks. In all conditions a central fixation point was visible throughout the trials. After a random inter-trial interval between 0.5 and 2.5 s a target appeared in the left or right periphery according to a pseudo-random sequence, and remained visible until the subject looked at it. Eccentricity and size of the target were varied between blocks leading to four different conditions: (1) big stimulus (3.1° × 13.2° rectangle) at high eccentricity (12.9°), (2) small stimulus (0.33° square) at high eccentricity, (3) big stimulus at small eccentricity (5°), and (4) small stimulus at small eccentricity (Figure 1 depicts these conditions). The order of blocks was randomized for each participant.

### 4.3 Procedure

Participants were seated at a distance of 65 cm from the computer screen. They completed a standard five-point calibration routine, consisting of a 1.8° white dot appearing in the center of the screen and moving to each of the corners of the screen, which took no longer than three minutes. After the calibration, four blocks—each containing one of the four types of target stimuli—were presented in random order with short breaks between blocks. Each block contained 100 trials, in which a fixation dot was visible on the center of the screen. After a random interval between 0.5 and 2.5 s an additional target appeared in the left or right periphery (counterbalanced) and remained visible until the participant looked at it. Participants were instructed to fixate at the central point, and look at each target as quickly as possible after it appeared. The next trial started once the eye-tracker had registered the participant’s fixation on the target. The entire experiment took approximately 30 min to complete.

### 4.4 Gaze-Contingent Eye-Tracking

During the experiment the eye-tracking data was accessed to monitor gaze positions. Whether a subject fixated on the initially presented central stimulus was determined by calculating the dispersion of measured gaze position from the center of the fixated object at the end of the random inter-trial interval. If a central fixation was registered for more than 20 samples (~330 ms), the peripheral target automatically appeared. If the subject looked at a peripheral stimulus, defined as the measured gaze position being in the area of the target stimulus for more than 20 samples (~330 ms), the stimulus automatically disappeared and the next trial began.

### 4.5 Eye-Tracking Data Analysis

After completing the experiment, the eye-tracking data was processed for all samples and analyzed for each trial using MATLAB (version 7.14.0.739, R2012a, 64bit), using previously established algorithms [15]. If eye-position data was missing in a sample, the data in this sample was interpolated with the average of the previous sample and the first subsequent successful sample. A refixation was defined as a horizontal change of gaze-position on the screen by more than 2.2 degrees of visual angle between two successive samples, with the onset being the time point before this change.

Trials involving noisy eye tracking data were excluded according to the following criteria: (1) if no gaze was registered on screen at trial onset, indicating that the eye tracker lost the signal, (2) if the trial contained too many excursions in fixation position (>20% of samples differed by more than 2.2 degree of visual angle from the previous sample) indicating fuzziness or signal-loss from the eye-tracker, or (3) if the first refixation occurred earlier than 0.1 s after the appearance of the peripheral target, as those refixations are probably unrelated to the appearance of the target (cf., [64]). Trials with the initial refixation to the wrong direction were registered as “misdirected refixations” and excluded from the analysis.

## Figures and Tables

**Table 1:** Means and standard deviations of refixation latencies (in ms) towards stimuli of different sizes and eccentricities.

		12.9° Eccentricity	5° Eccentricity
0.33° target	Mean	275	272
	SD	37.9	52.1
3.1° × 13.2° target	Mean	261	290
	SD	45.1	51.1

## Supplementary Materials

Supplementary materials including the full dataset are available online at http://www.mdpi.com/2411-5150/1/4/25/S1.
